# Microsatellite instability in human testicular germ cell tumours.

**DOI:** 10.1038/bjc.1995.387

**Published:** 1995-09

**Authors:** R. A. Huddart, R. Wooster, A. Horwich, C. S. Cooper

**Affiliations:** Section of Molecular Carcinogenesis, Institute of Cancer Research, Sutton, Surrey, UK.

## Abstract

**Images:**


British Journal of Cancer (1995) 72, 642-645

© 1995 Stockton Press All rights reserved 0007-0920/95 $9.00

SHORT COMMUNICATION

### Microsatellite instability in human testicular germ cell tumours

RA Huddart[1], R Wooster[1], A Horwich[2] and CS Cooper[1]

[1]*Section of Molecular Carcinogenesis, Institute of Cancer Research, Sutton, Surrey SM2 5NG, UK;* [2]*Academic Unit of Radiotherapy and Oncology, Royal Marsden Hospital, Sutton, Surrey SM2 5NR, UK.*

**Summary** DNA samples obtained from 29 testicular germ cell tumours have been screened for instability at nine different microsatellite sequences consisting of dinucleotide, trinucleotide and tetranucleotide loci. Overall, in tumours from six (21%) patients we found abnormalities in at least one of the loci examined. Mutation was most frequently found in tetranucleotide and trinucleotide repeats with only a low proportion of alterations in dinucleotide repeats. This pattern of instability is distinct from that reported in colorectal cancer and other cancers that have a high level of alterations in dinucleotide repeats.

**Keywords:** testicular germ cell tumours; microsatellite instability

Testicular germ cell tumours (TGCTs) form a histologically heterogeneous group of neoplasms that are thought to arise from premeiotic germ cells. Despite receiving considerable attention in recent years very little is known about the molecular mechanism of TGCT development. Cytogenetic studies have identified isochromosome 12p as a consistent karyotypic abnormality that is present in the majority of tumours (Atkin and Baker, 1982). Loss of heterozygosity studies have identified multiple chromosomal regions that may be the sites of tumour-suppressor genes (Murty *et al.*, 1994*a*) while H-*RAS*, N-*RAS* and *DCC* abnormalities have all been implicated in the aetiology of these tumours (Ganguly *et al.*, 1990; Moul *et al.*, 1992; Murty *et al.*, 1994*b*).

Several studies have implicated defects in DNA mismatch repair in the pathogenesis of hereditary non-polyposis colorectal cancer (HNPCC). Widespread alterations in genomic DNA, as indicated by changes in microsatellite sequences consisting of dinucleotide and trinucleotide repeats and in other simple repeat sequences, were discovered in colorectal tumours (Armour *et al.*, 1989; Aaltonen *et al.*, 1993; Ionov *et al.*, 1993; Thibodeau *et al.*, 1993). Recognition that the types of genetic alterations observed in these studies were similar to those associated with abnormalities of bacterial mismatch repair genes such as *mutS* and *mutL* initially led to the discovery that the human homologues of these genes, *hMSH2* and *hMLH1* (Fishel *et al.*, 1993; Leach *et al.*, 1993; Bronner *et al.*, 1994; Papadopoulous *et al.*, 1994), were mutated in HNPCC kindreds. More recently mutations in two human homologues of yeast repair genes designated *PMS1* and *PMS2* have also been found in HNPCC families (Nicolaides *et al.*, 1994). In addition to the observed abnormalities in colorectal cancer, a high frequency of genetic instability of microsatellites (the mutator phenotype) has also been observed in other classes of human cancer, including endometrial (Risinger *et al.*, 1993; Tucker Burks *et al.*, 1994) pancreatic (Han *et al.*, 1993), gastric (Han *et al.*, 1993) oesophageal (Meltzer *et al.*, 1994) and small-cell lung cancer (Merlo *et al.*, 1994; Shridhar *et al.*, 1994). In contrast, lower frequencies of instability have been observed in breast cancers, ovarian cancers, soft-tissue sarcomas and tumours of the nervous system (Wooster *et al.*, 1994). In the present study we have assessed the stability of microsatellite sequences in TGCTs.

Correspondence: RA Huddart

Received 27 February 1995; revised 19 April 1995; accepted 21 April 1995

### Materials and methods

Tumour and blood samples were collected from testicular cancer patients at the Royal Marsden Hospital, Sutton, UK. DNA was prepared from tumour and matched lymphocyte specimens as described previously (Sambrook *et al.*, 1989; Lahiri and Nurnberger, 1991). Short tandem repeats were amplified using polymerase chain reaction (PCR). In each case one primer was radiolabelled with $^{32}P$ at the 5′-end using T4 polynucleotide kinase and [$\gamma$-$^{32}P$]ATP. PCR was performed in at 25 µl volume containing 50 ng of DNA, 2.5 µl of 10 × reaction buffer (Advanced Biotechnology), 0.2 mM of each dNTP, 0.1 unit of *Taq* polymerase and 20 pmol of each primer. PCR was performed using cycle conditions of 94°C for 60 s, 55-60°C for 60 s and 72°C for 60 s. Following amplification for 35 cycles the products were denatured in formamide-EDTA loading buffer and subjected to electrophoresis in denaturing polyacrylamide gels (6%, w v). Visualisation was by autoradiograph for 5-72 h. Nine polymorphic loci were examined. Seven of these markers were the same as used by Wooster *et al.* (1994), to allow comparison between tumour types. D1S216 was substituted for chromosome 16 markers to widen the number of chromosomes examined and to examine an area where instability had been previously reported (Murty *et al.*, 1994*c*). TFIID was included as a trinucleotide marker which we had found to be useful in screening for a low rate of instability in other tumours. Primers used to amplify the myotonic dystrophy CAG repeat and the androgen receptor CAG repeat are reported by Wooster *et al.* (1994). The primers that amplify the von Willebrand's factor tetranucleotide repeats vWFa (Kimpton *et al.*, 1992) and vWFb (van Amstel and Reisman 1990) and to amplify four dinucleotide repeats corresponding to the loci *D1S216*, *D2S123*, *D16S303* and *D17S5796* (Thompson *et al.*, 1992; Weissenbach *et al.*, 1992) are found in the relevant references. The primers used to amplify the TFIID trinucleotide repeat are 5′-TGCCACTGGACTGACC-3′ and 5′-GCTGCCACTGCCTGTT-3′.

### Results

In the present study a series of 29 testicular germ cell tumours were examined for instability in a series of nine tandem repeats. The tumour group consisted of 14 seminomas, 12 non-seminomatous germ cell tumours (NSGCTs) and four tumours with combined seminoma-NSGCT histology. The loci examined included two tetranucleotide repeats, vWFa and vWFb, located within introns of the gene encoding von Willebrand's factor (vWF) and three trinucleotide repeats present in genes for the androgen receptor (AR), myotronic dystrophy (DM) and the TFIID transcription factor. Four dinucleotide repeats (*D1S216, D2S123, D16S303* and *D17S796*) were also examined. Alleles amplified by PCR frequently exhibited a characteristic stutter appearing as doublets with the bands of different or, in some cases, similar intensity. Abnormal sized bands were detected in six different tumours (Table I, Figure 1). To exclude technical artefacts or specimen contamination these abnormalities were reproduced in independent PCR amplifications and separate gel loadings. The consistent occurrence of identical alleles with the same size in normal and tumour DNA at other microsatellite loci excluded errors such as incorrect numbering or tissue contamination.

Three of the tumours exhibited a single abnormality with two tumours containing two and a single tumour containing four alterations (Table I). Notably the highest frequency of abnormality was observed for tetranucleotide repeats and trinucleotide repeats (10 of 145 typings, 7%) while there was a relatively low level of alteration in dinucleotide repeats (1 of 116 typings, 0.9%). There was no clear association with particular histological type as abnormalities were detected in seminomas, in a NSGCT and in a tumour with combined histology.

### Discussion

In an unselected group of primary testicular cancers six (21%) tumours showed microsatellite instability detected as mobility shift in tumour DNA compared with paired lymphocyte DNA. The highest level of abnormalities was found in tetranucleotide and trinucleotide repeats with only a low level of alteration in dinucleotide repeats. This situation contrasts with that found for colorectal and other classes of human cancer where a high level of mutations are found in dinucleotide repeats. Indeed the spectrum of mutation resembles more closely that reported by Wooster *et al.* (1994) who, in a study of 196 breast cancers, ovarian cancers, soft tissue sarcomas and tumours of the nervous system, also detected the majority of alterations in tri- and tetranucleotide

**Figure 1** Analysis of microsatellite repeats in paired lymphocyte (B) and tumour (T) DNAs from the same patient. The GCT number assigned to each patient with a testicular germ cell tumour is also shown. Instabilities were observed in the repeat found within the TFIID transcription factor gene (TFIID), the CAG repeat within the androgen receptor gene (AR), the TCTA tetranucleotide repeats found within introns of the von Willebrand's factor gene (*vWFa, vWFb*) and the CA dinucleotide repeat at the *D16S303* locus. Alleles amplified by PCR are frequently associated with a characteristic stutter and appear as doublets with bands of different or similar intensity. Additional alleles detected in tumours are indicated by small arrows.

**Table I** Testicular germ cell tumours with microsatellite instabilities

| Patient | Histology[a] | Age | Altered loci[b] | Altered repeat Tetranucleotide | Trinucleotide | Dinucleotide |
|---|---|---|---|---|---|---|
| GCT1 | Sem | 46 | 1 | *vWFb* | | |
| GCT3 | Sem | 45 | 4 | *vWFb, vWFa* | *TFIID, AR* | |
| GCT5 | NSGCT | 46 | 2 | *vWFa* | *TFIID* | |
| GCT7 | Sem | 27 | 2 | | *TFIID* | *D16S303* |
| GCT10 | Comb | 33 | 1 | *vWFa* | | |
| GCT28 | Sem | 47 | 1 | *vWFb* | | |

[a]Sem, seminomas; NSGCT, non-seminomatous germ cell tumours; Comb, combined tumour exhibiting characteristics of both seminomas and NSGCT. [b]A total of four dinucleotide, three trinucleotide and two tetranucleotide microsatellite repeats were examined for instability in each tumour as described in Materials and methods. [c]vWFa and vWFb are two loci within introns of the van Willebrand factor gene; AR, androgen receptor microsatellite; TFIID, a microsatellite repeat within the TFIID transcription factor gene.

repeats. The mutation rate in tri- and tetranucleotide repeats in TGCT (10 in 145 typings, 7%) was, however, somewhat higher than that reported by Wooster *et al.* (1994), who found 15 alterations in 980 typings (1.5%). Although stability of tri- and tetranucleotide repeats has in general been studied less thoroughly than dinucleotide repeats, instability of trinucleotide repeats has been detected in colorectal tumours which have the mutator phenotype (Aaltonen *et al.* (1993). Abnormalities in trinucleotide repeats have also been detected in a variety of human genetic conditions. For example, CAG repeats or CTG repeats are expanded in myotronic dystrophy, X-linked spinobulbar muscular atrophy, Huntington's disease and spinocerebellar ataxia type 1 (Willems, 1994).

Our results are in agreement with those obtained by Lothe *et al.* (1993), who failed to find replication errors in a series of 86 germ cell tumours, including familial cases, using seven dinucleotide repeat markers mapping to 1p, 5q, 8p, 10p, 11p, 13 and 17q. A high level of replication error type genetic instability was observed in germ cell tumours by Murty *et al.* (1994*c*). However, the instability was restricted to markers at chromosome 1q42–43 and the abnormalities included loss of heterozygosity, dinucleotide repeat replication errors as well as alterations of variable number of tandem repeat regions. In parallel experiments in the same study alterations were not observed in dinucleotide repeats mapping to 12q, 17p and 18q. In contrast, in the present study we show that replication errors are not restricted to chromosome 1 but can occur in di-, tri- and tetranucleotide repeats mapping to chromosomes 6 (*TFIID*), 12 (*vWF*), 16 (*D16S303*) and X (*AR*).

The significance of the microsatellite instability found in TGCTs needs further investigation. The rarity of dinucleotide repeat abnormalities would argue against involvement of the *hMSH2* and *hMLH1* genes. However it is possible that mutation in other repair genes could be responsible for the abnormalities in tri- and tetranucleotide repeats. As more information becomes available on these genes it would therefore be of interest to investigate their role in the aetiology of TGCT. In this regard it is worthy of note that the karyotypes of TGCTs are frequently complex with a DNA index commonly in the hypotriploid–hypertriploid range while high levels of loss of heterozygosity are observed for many chromosomal arms including 2p, 3p, 3q, 5p, 9p, 9q, 10q, 11p, 12q, 13q, 17p, 17q, 18p, 18q and 20p (Murty *et al.*, 1994*a*). Though other mechanisms may also be acting both of these findings would be consistent with the presence of mutations in repair genes that predispose to larger genetic changes in TGCT.

**Acknowledgements**
We thank Christine Bell for typing this manuscript and Dr M Stratton, Dr P Karran and Professor R Lahue for helpful discussion. This work is funded by grants from the Cancer Research Campaign. RH is supported by a CRC Clinical Research Fellowship.

**References**

AALTONEN LA, PELTOMAKI P, LEACH FS, SISTONEN P, PYLKKANEN L, MECKLIN J-P, JARVINEN H, POWELL SM, JEN J, HAMILTON SR, PETERSON GM, KINZLER KW, VOGELSTEIN B AND DE LA CHAPELLE A. (1993). Clues to the pathogenesis of familial colorectal cancer. *Science*, **260**, 812–816.

ARMOUR JAL, PATEL I, THEIN S, FEY MF AND JEFFREYS AJ. (1989). Analysis of somatic mutations of human minisatellite loci in tumours and cell lines. *Genomics*, **4**, 328–334.

ATKIN NB AND BAKER MC. (1982). Specific chromosome change, i(12p) in testicular tumours. *Lancet*, **2**, 1349.

BRONNER CE, BAKER SM, MORRISON PT, WARREN G, SMITH LG, LESCOE MK, KANE M, EARABINO C, LIPFORD J, LINDBLOM A, TANNERGARD P, BOLLAG RJ, GOODWIN AR, WARD DC, NORDENSKJOD M, FISHEL R, KOLODNER R AND LISKAY RM. (1994). Mutation in the DNA mismatch repair gene homologue hMLH1 is associated with hereditary non polyposis colon cancer. *Nature*, **368**, 258–261.

FISHEL R, LESCOE MK, RAO MRS, COPELAND NG, JENKINS NA, GARBER J, KANE M AND KOLODNER R. (1993). The human mutator gene homology MSH2 and its association with hereditary nonpolyposis colon cancer. *Cell*, **75**, 1027–1038.

GANGULY S, MURTY VVVS, SAMANIEGO F, REUTER VE, BOSL GJ AND CHAGANTI RSK. (1990). Detection of preferential NRAS mutations in human male germ cell tumours by the polymerase chain reaction. *Genes Chrom. Cancer*, **1**, 228–232.

HAN HJ, YANAGISAWA A, KATO Y, PARK J-G AND NAKAMURA Y. (1993). Genetic instability in pancreatic cancer and poorly differentiated type of gastric cancer. *Cancer Res.*, **53**, 5087–5089.

IONOV Y, PIENADO MA, MALKHOSYAN S, SHIBATA D AND PERUCHO M. (1993). Ubiquitous somatic mutations in simple repeated sequences reveal a new mechanism for colonic carcinogenesis. *Nature*, **363**, 558–561.

KIMPTON C, WALTON A AND GILL P. (1992). A further tetranucleotide repeat polymorphism in the vwf gene. *Hum. Mol. Genet.*, **1**, 287.

LAHIRI DK AND NURNBERGER JI. (1991). A rapid non-enzymatic method for the preparation of HMW DNA from blood for RFLP studies. *Nucleic Acids Res.*, **19**, 5444.

LEACH FS, NICOLAIDES NC, PAPADOPOULOUS N, LIU B, JEN J, PARSONS R, PELTOMAKI R, SISTONEN P, AALTONEN LA, NYSTROM-LAHTI M, GUAN X-Y, ZHANG J, MELTZER PS, YU J-W, KAO F-T, CHEN DJ, CEROSALETTI KM, FOURNIER RE, TODD S, LEWIS T, LEACH RJ, NAYLOR SL, WEISSENBACH J, MECKLIN J-P, JARVINEN H, PETERSEN GM, HAMILTON SR, GREEN J, JASS J, WATSON P, LYNCH HT, TRENT JM, DE LA CHAPELLE A, KINZLER KW AND VOGELSTEIN B. (1993). Mutation of a mutS homology in hereditary nonpolyposis colorectal cancer. *Cell*, **75**, 1215–1225.

LOTHE RA, PELTOMAKI P, MELING GI, AALTONEN LA, NYSTROM-LAHTI M, PYLKKANEN L, HEIMDAL K, ANDERSON TI, MOLLER P, ROGNUM TO, FOSSA SD, HALDORSEN T, LANGMARK F, BROGGER A, DE LA CHAPELLE A AND BORRESEN AL. (1993). Genomic instability in colorectal cancer: relationship to clinicopathological variables and family history. *Cancer Res.*, **53**, 5849–5852.

MELTZER SJ, YIN J, MANIN B, RHYU M-G, COTTRELL J, HUDSON E, REDD JL, KRASNA MJ, ABRAHAM JM AND REID BJ. (1994). Microsatellite instability occurs frequently and in both diploid and aneuploid cell populations of Barrett's associated esophogeal adenocarcinomas. *Cancer Res.*, **54**, 3379-3382.

MERLO A, MABRY M, GABRIELSON E, VOLLMER R, BAYLIN SB AND SIDRANSKY D. (1994). Frequent microsatellite instability in primary small cell lung cancer. *Cancer Res.*, **54**, 2098-2101.

MOUL JW, THEUNE SM AND CHANG EH. (1992). Detection of RAS mutation in archival testicular germ cell tumours by polymerase chain reaction and oligonucleotide hybridization. *Genes Chrom. Cancer*, **5**, 109-118.

MURTY VVVS, BOSL GJ, HOULDSWORTH J, MEYERS M, MUKHERJEE AB, REUTER V AND CHAGANTI RSK. (1994*a*). Allelic loss and somatic differentiation in human male germ cell tumours. *Oncogene*, **9**, 2245-2251.

MURTY VVVS, LI RG, HOULDSWORTH J, BRONSON DL, REUTER VE, BOSL GJ AND CHAGANTI RSK. (1994*b*). Frequent allelic deletion and loss of expression characterize the DCC gene in male germ cell tumours. *Oncogene*, **9**, 3227-3231.

MURTY VVVS, LI R-G, MATTHEW S, REUTER VE, BRONSON DL, BOSL GJ AND CHAGANTI RSK. (1994*c*). Replication error type genetic instability of 1q42-43 in human male germ cell tumours. *Cancer Res.*, **54**, 3983-3985.

NICOLAIDES NC, PAPADOPOULOS N, LIU B, WEI Y-F, CARTER KC, RUBEN SN, ROSEN CA, HASELTINE WA, FLEISCHMANN RD, FRASER CM, ADAMS MD, VENTER JC, DUNLOP MD, HAMILTON SR, PETERSEN GM, DE LA CHAPPELLE A, VOGELSTEIN B AND KINZLER KW. (1994). Mutations of two PMS homologues in hereditary nonpolyposis colon cancer. *Nature*, **371**, 75-80.

PAPADOPOULOUS N, NICOLAIDES NC, WEI Y-F, RUBEN SM, CARTER KC, ROSEN CA, HASELTINE WA, FLEISHMAN RD, FRASER CM, ADAMS MD, VENTER JC, HAMILTON SR, PETERSON GM, WATSON P, LYNCH HT, PELTOMAKI P, MECKLIN J-P, DE LA CHAPELLE A, KINZLER KW AND VOGELSTEIN B. (1994). Mutation of a mutL homology in hereditary colon cancer. *Nature*, **263**, 1625-1629.

RISINGER JI, BERCHUCK A, KOHLER MF, WATSON P, LYNCH HT AND BOYD J. (1993). Genetic instability of microsatellites in endometrial carcinoma. *Cancer Res.*, **53**, 5100-5103.

SAMBROOK J, FRITSCH EF, MANIATIS T. (1989). *Molecular Cloning*. Cold Spring Harbor Laboratory Press: Cold Spring Harbor, NY.

SHRIDHAR V, SIEGFRIED J, HUNT J, DEL MAR ALONSO M AND SMITH DI. (1994). Genetic instability of microsatellite sequences in many non-small cell lung carcinomas. *Cancer Res.*, **54**, 2084-2087.

THIBODEAU SN, BREN G AND SCHAD D. (1993). Microsatellite instability in cancer of the proximal colon. *Science*, **260**, 816-819.

THOMPSON AD, SHEN Y, HOLMAN K, SUTHERLAND GR, CALLEN DF AND RICHARDS RI. (1992). Isolation and characterization of (AC)n microsatellite genetic markers from human chromosome 16. *Genomics*, **13**, 402-408.

TUCKER BURKS R, KESSIS TD, CHO KR AND HENDRICK L. (1994). Microsatellite instability in endometrial carcinoma. *Oncogene*, **9**, 1163-1166.

VAN AMSTEL HK AND RIESMAN PH. (1990). Tetranucleotide repeat polymorphism in the vwf gene. *Nucleic Acids Res.*, **18**, 4957.

WEISSENBACH J, GYAPAY G, DIB C, VIGNAL A, MORISSETTE J, MILLASSEAU P, VAYSSEIX G, LATHROP M. (1992). A second generation linkage map of the human genome. *Nature*, **359**, 794-801.

WILLEMS PJ. (1994). Dynamic mutations hit double figures. *Nature Genet.*, **8**, 213-215.

WOOSTER R, CLETON-JANSON A-M, COLLINS N, MANGION J, CORNELIS RS, COOPER CS, GUSTERSON BA, PONDER BAJ, VON DEIMLING A, WIESTLER OD, CORNELISSE CJ, DEVILEE P AND STRATTON MR. (1994). Instability of short tandem repeats microsatellites in human cancers. *Nature Genet.*, **6**, 151-156.


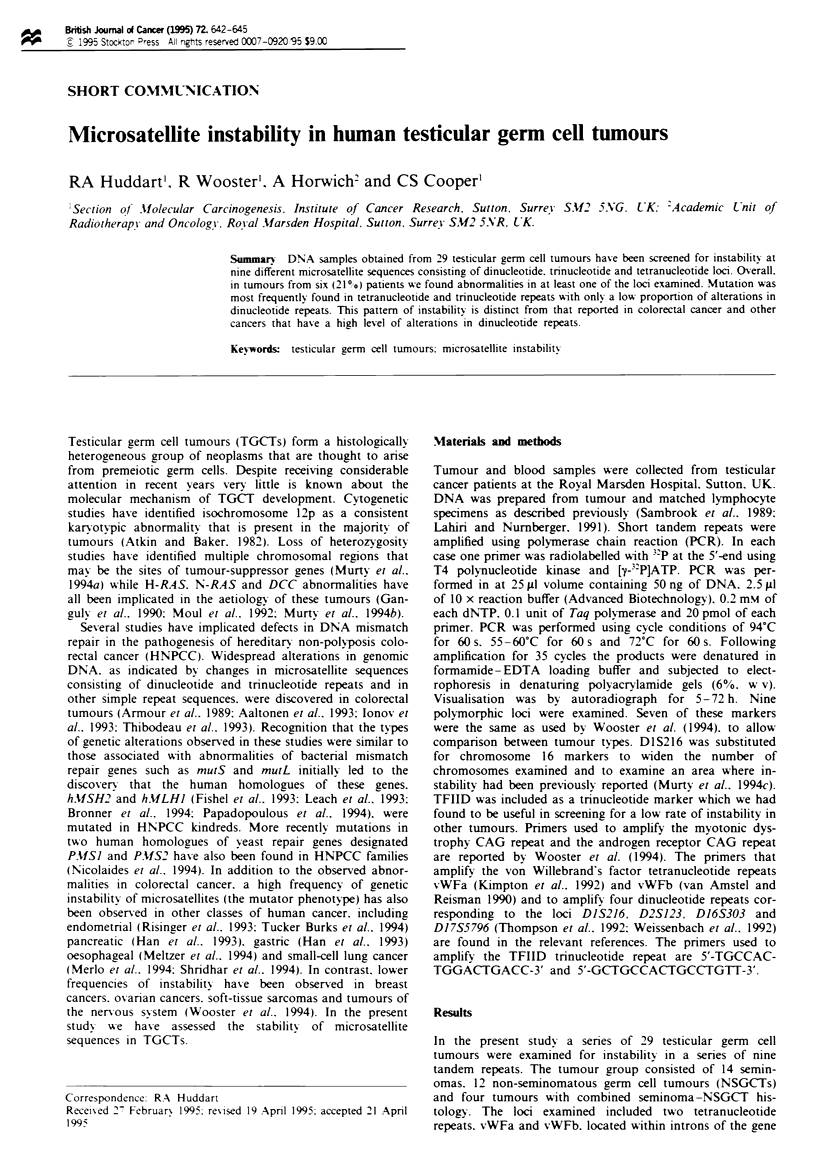

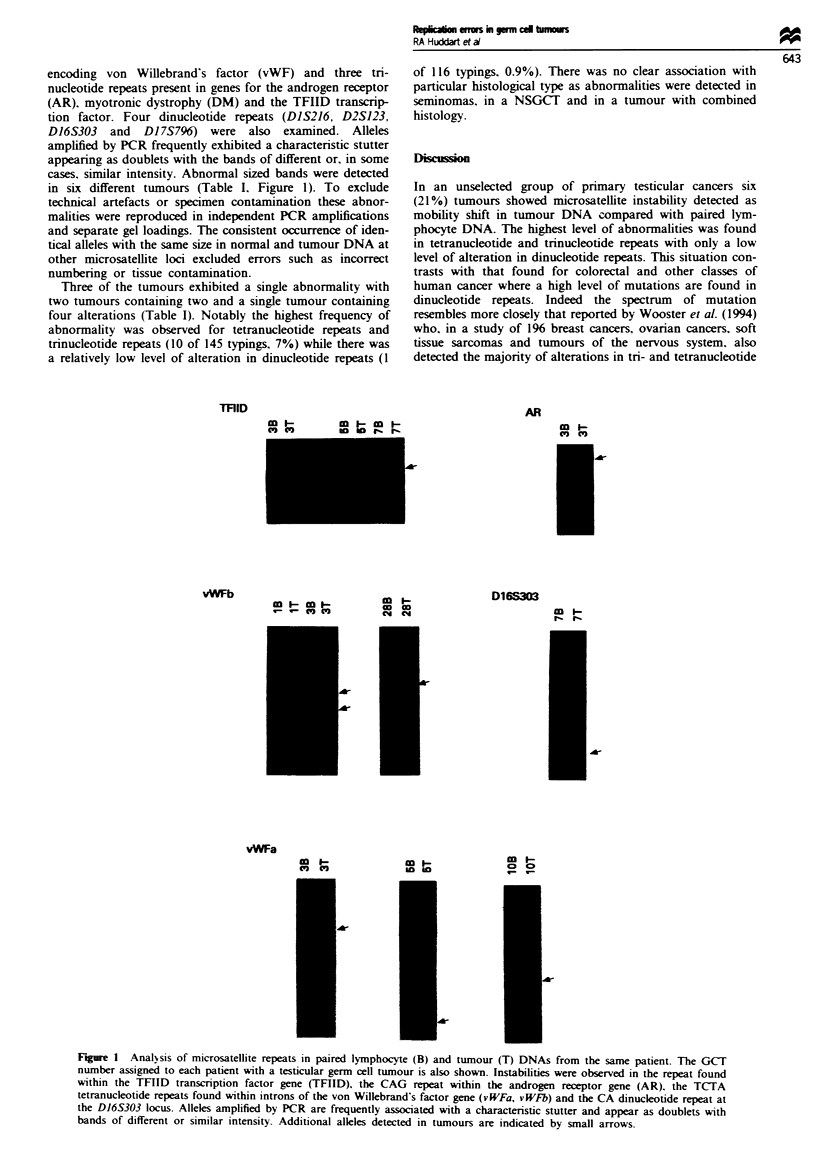

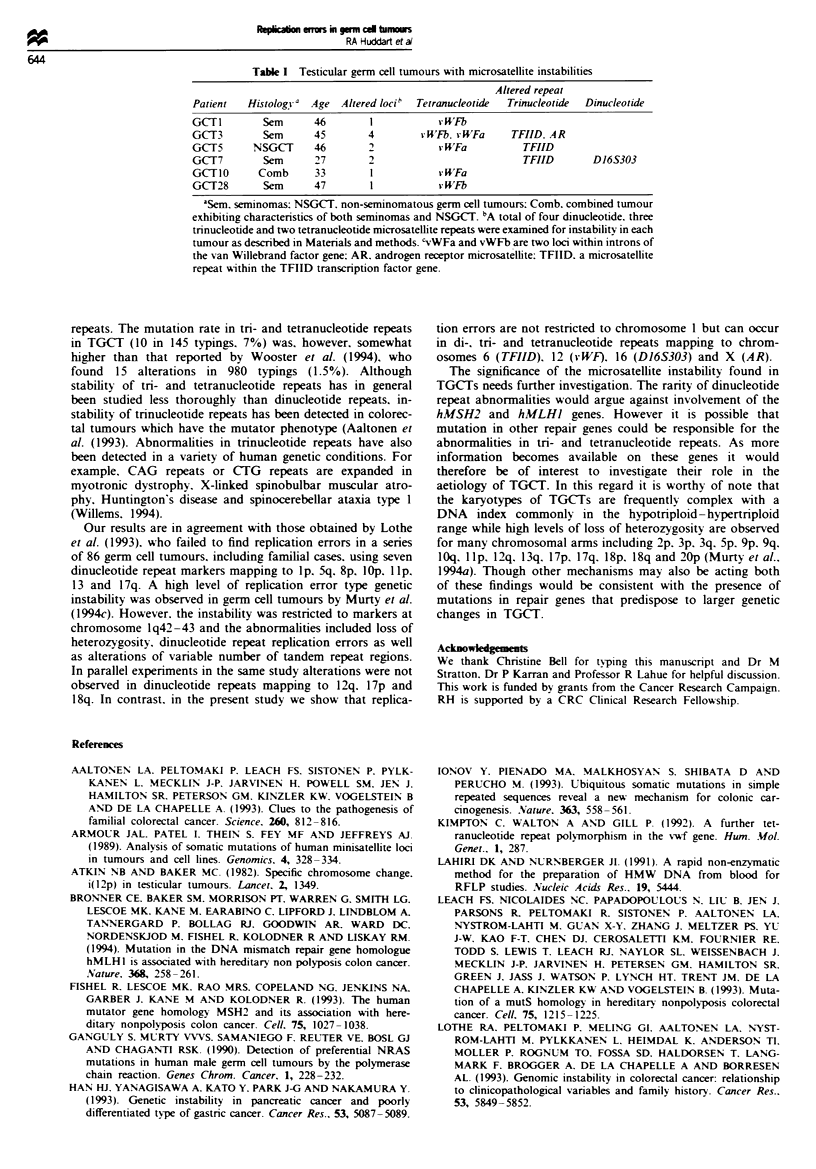

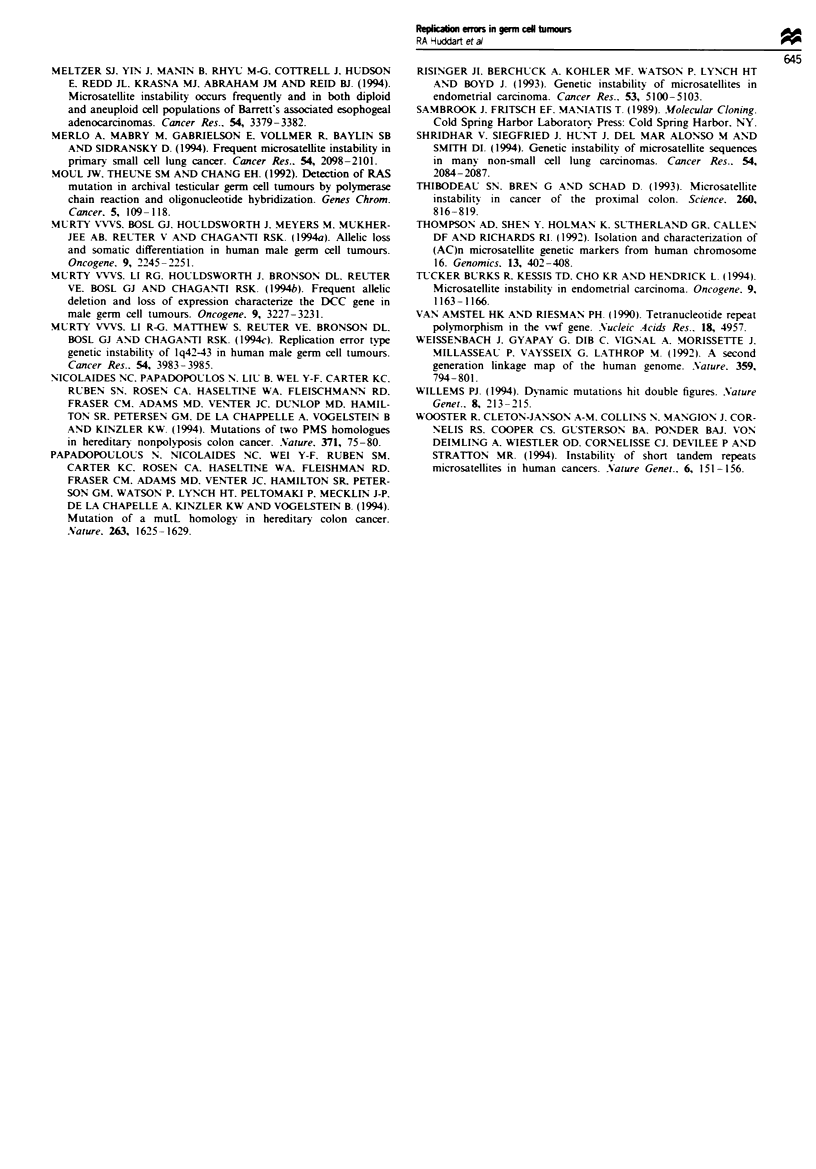


## References

[OCR_00309] Aaltonen L. A., Peltomäki P., Leach F. S., Sistonen P., Pylkkänen L., Mecklin J. P., Järvinen H., Powell S. M., Jen J., Hamilton S. R. (1993). Clues to the pathogenesis of familial colorectal cancer.. Science.

[OCR_00318] Armour J. A., Patel I., Thein S. L., Fey M. F., Jeffreys A. J. (1989). Analysis of somatic mutations at human minisatellite loci in tumors and cell lines.. Genomics.

[OCR_00323] Atkin N. B., Baker M. C. (1982). Specific chromosome change, i(12p), in testicular tumours?. Lancet.

[OCR_00471] Burks R. T., Kessis T. D., Cho K. R., Hedrick L. (1994). Microsatellite instability in endometrial carcinoma.. Oncogene.

[OCR_00337] Fishel R., Lescoe M. K., Rao M. R., Copeland N. G., Jenkins N. A., Garber J., Kane M., Kolodner R. (1993). The human mutator gene homolog MSH2 and its association with hereditary nonpolyposis colon cancer.. Cell.

[OCR_00343] Ganguly S., Murty V. V., Samaniego F., Reuter V. E., Bosl G. J., Chaganti R. S. (1990). Detection of preferential NRAS mutations in human male germ cell tumors by the polymerase chain reaction.. Genes Chromosomes Cancer.

[OCR_00346] Han H. J., Yanagisawa A., Kato Y., Park J. G., Nakamura Y. (1993). Genetic instability in pancreatic cancer and poorly differentiated type of gastric cancer.. Cancer Res.

[OCR_00351] Ionov Y., Peinado M. A., Malkhosyan S., Shibata D., Perucho M. (1993). Ubiquitous somatic mutations in simple repeated sequences reveal a new mechanism for colonic carcinogenesis.. Nature.

[OCR_00359] Kimpton C., Walton A., Gill P. (1992). A further tetranucleotide repeat polymorphism in the vWF gene.. Hum Mol Genet.

[OCR_00367] Lahiri D. K., Nurnberger J. I. (1991). A rapid non-enzymatic method for the preparation of HMW DNA from blood for RFLP studies.. Nucleic Acids Res.

[OCR_00382] Lothe R. A., Peltomäki P., Meling G. I., Aaltonen L. A., Nyström-Lahti M., Pylkkänen L., Heimdal K., Andersen T. I., Møller P., Rognum T. O. (1993). Genomic instability in colorectal cancer: relationship to clinicopathological variables and family history.. Cancer Res.

[OCR_00393] Meltzer S. J., Yin J., Manin B., Rhyu M. G., Cottrell J., Hudson E., Redd J. L., Krasna M. J., Abraham J. M., Reid B. J. (1994). Microsatellite instability occurs frequently and in both diploid and aneuploid cell populations of Barrett's-associated esophageal adenocarcinomas.. Cancer Res.

[OCR_00401] Merlo A., Mabry M., Gabrielson E., Vollmer R., Baylin S. B., Sidransky D. (1994). Frequent microsatellite instability in primary small cell lung cancer.. Cancer Res.

[OCR_00405] Moul J. W., Theune S. M., Chang E. H. (1992). Detection of RAS mutations in archival testicular germ cell tumors by polymerase chain reaction and oligonucleotide hybridization.. Genes Chromosomes Cancer.

[OCR_00411] Murty V. V., Bosl G. J., Houldsworth J., Meyers M., Mukherjee A. B., Reuter V., Chaganti R. S. (1994). Allelic loss and somatic differentiation in human male germ cell tumors.. Oncogene.

[OCR_00418] Murty V. V., Li R. G., Houldsworth J., Bronson D. L., Reuter V. E., Bosl G. J., Chaganti R. S. (1994). Frequent allelic deletions and loss of expression characterize the DCC gene in male germ cell tumors.. Oncogene.

[OCR_00421] Murty V. V., Li R. G., Mathew S., Reuter V. E., Bronson D. L., Bosl G. J., Chaganti R. S. (1994). Replication error-type genetic instability at 1q42-43 in human male germ cell tumors.. Cancer Res.

[OCR_00430] Nicolaides N. C., Papadopoulos N., Liu B., Wei Y. F., Carter K. C., Ruben S. M., Rosen C. A., Haseltine W. A., Fleischmann R. D., Fraser C. M. (1994). Mutations of two PMS homologues in hereditary nonpolyposis colon cancer.. Nature.

[OCR_00444] Risinger J. I., Berchuck A., Kohler M. F., Watson P., Lynch H. T., Boyd J. (1993). Genetic instability of microsatellites in endometrial carcinoma.. Cancer Res.

[OCR_00454] Shridhar V., Siegfried J., Hunt J., del Mar Alonso M., Smith D. I. (1994). Genetic instability of microsatellite sequences in many non-small cell lung carcinomas.. Cancer Res.

[OCR_00458] Thibodeau S. N., Bren G., Schaid D. (1993). Microsatellite instability in cancer of the proximal colon.. Science.

[OCR_00466] Thompson A. D., Shen Y., Holman K., Sutherland G. R., Callen D. F., Richards R. I. (1992). Isolation and characterisation of (AC)n microsatellite genetic markers from human chromosome 16.. Genomics.

[OCR_00480] Weissenbach J., Gyapay G., Dib C., Vignal A., Morissette J., Millasseau P., Vaysseix G., Lathrop M. (1992). A second-generation linkage map of the human genome.. Nature.

[OCR_00484] Willems P. J. (1994). Dynamic mutations hit double figures.. Nat Genet.

[OCR_00488] Wooster R., Cleton-Jansen A. M., Collins N., Mangion J., Cornelis R. S., Cooper C. S., Gusterson B. A., Ponder B. A., von Deimling A., Wiestler O. D. (1994). Instability of short tandem repeats (microsatellites) in human cancers.. Nat Genet.

[OCR_00474] van Amstel H. K., Reitsma P. H. (1990). Tetranucleotide repeat polymorphism in the vWF gene.. Nucleic Acids Res.

